# PPARα-Mediated Fatty Acid Catabolism in Astrocytes Was Involved in Improvement of Cognitive Dysfunction by Phlorizin in APP/PS1 Mice

**DOI:** 10.3390/antiox14111321

**Published:** 2025-10-31

**Authors:** Yan Fu, Xuya Zhang, Lingling Li, Hong Jiang, Qiaozhi Ren, Tianxing Yi, Yali Zhang, Yi Lu

**Affiliations:** School of Traditional Chinese Medicine, Beijing University of Chinese Medicine, Beijing 100105, China; 20230931133@bucm.edu.cn (Y.F.); 20220931137@bucm.edu.cn (X.Z.); 20240941084@bucm.edu.cn (L.L.); 20240931136@bucm.edu.cn (H.J.); 20240931133@bucm.edu.cn (Q.R.); 20240941083@bucm.edu.cn (T.Y.); 20210931137@bucm.edu.cn (Y.Z.)

**Keywords:** Alzheimer’s disease, lipid metabolic dysfunction, cognitive dysfunction, phlorizin, astrocyte

## Abstract

Central lipid metabolism disorders are crucial for the development of Alzheimer’s disease (AD). Phlorizin (PHZ) improved lipid metabolism abnormalities in AD nematodes, but its mechanism of action in improving AD-related symptoms and whether it can alleviate AD cognitive impairment remain unclear. To elucidate the effects and mechanisms of PHZ on lipid metabolism disorders in an AD model, gavage administration of PHZ for 8 weeks improved cognitive dysfunction and lipid disorders in APPswe/PSEN1dE9 (APP/PS1) mice. Concurrently, in astrocytes induced by palmitic acid (PA)- mediated lipid metabolic disorder, PHZ treatment improved astrocytic lipid accumulation by upregulating the target peroxisome proliferator-activated receptor α (PPARα) and its downstream pathways, thereby promoting astrocytic fatty acid oxidation. We validated PHZ’s strong in vitro binding affinity with PPARα. Co-culture systems of lipid-metabolically disordered astrocytes and neurons further demonstrated that PHZ significantly improved neuronal cell viability and reduced intracellular lipid accumulation, thereby decreasing the expression of enzymes associated with β-amyloid protein (Aβ) production. This study demonstrates that gavage administration of PHZ for 2 months improves cognitive deficits and pathological markers in AD mice. Furthermore, at the cellular level, PHZ may exert its effects by enhancing astrocytic lipid metabolism, thereby preventing neuronal lipotoxicity and mitigating AD progression.

## 1. Introduction

Alzheimer’s disease (AD) is a severe neurodegenerative disease with cognitive dysfunction and behavioral disorders [1], which mainly occurs in the elderly [2]. There is currently no effective cure for AD [3]. Although abnormal accumulation of lipids was described in the first reports of AD neuropathology [4], it is only in recent decades that dysregulation of lipid homeostasis has become a focus of AD research [5]. In the clinical setting, lipidomic and metabolomic studies have consistently demonstrated changes in the levels of various lipid classes that occur in the early stages of the AD brain [6]. Available studies [5] suggest that the brain is heavily dependent on astrocyte mitochondrial β-oxidation to degrade fatty acids (FAs) and maintain lipid homeostasis [7], and that aberrant lipid metabolism induces the accumulation of lipid droplets (LDs) [8] and subsequent neurodegeneration [9]. PPARs are core transcription factors that enable cells to sense fatty acid levels and regulate the expression of genes involved in fatty acid metabolism [10,11]. When intracellular levels of fatty acids (or their derivatives) increase [12], they act as ligands to activate PPARs [13]. Activated PPARα translocates to the nucleus [14], binds to specific regions on DNA, and thereby initiates or enhances the transcription of a series of genes related to fatty acid metabolism. Direct downstream target genes of PPARα include acyl-CoA synthetase (ACS) and carnitine palmitoyltransferase 1A (CPT1A) [12]. In other words, PPARα promotes fatty acid β-oxidation (FAO) and the tricarboxylic acid cycle by up-regulating the gene expression of ACS and CPT1A [15]. The mitochondrial FAO cycle involves four key dehydrogenase enzymes: short-chain acyl-CoA dehydrogenase (ACADS), medium-chain acyl-CoA dehydrogenase (ACADM), long-chain acyl-CoA dehydrogenase (ACADL), and extremely long-chain acyl-CoA dehydrogenase (ACADVL) [16]. Among these, ACADL and ACADVL constitute the rate-limiting steps of FAO [17], responsible for the initial dehydrogenation of long-chain (C12-C18) and extremely long-chain (C18-C22) fatty acids [18], respectively. Furthermore, the downregulation of their activity represents the most pronounced and devastating alteration in AD [19]. Long-chain fatty acids serve as critical backup fuel for the brain during glucose shortages. Inhibition of ACADL/VL prevents substantial long-chain fatty acids from entering β-oxidation for ATP production, rendering neurons more vulnerable under energy stress [20]. Mitochondrial fatty acid oxidation disorders constitute an expanding group of metabolic pathologies. Impaired function of ACADs—particularly ACADL and ACADVL—represents a pivotal link connecting mitochondrial dysfunction [21], energy metabolism crises, and lipid toxicity in the AD brain [22]. These enzymes not only reflect metabolic network failure but also actively participate in and amplify neurodegenerative processes in AD [23].

Phlorizin (PHZ), a natural dihydrochalcone polyphenol [24], improves glycolipid metabolism and possesses potent antioxidant activity [25]. Its antioxidant effect [26], primarily achieved by scavenging free radicals, protects cells from oxidative damage [27]. Consequently, PHZ plays a key role in slowing cellular aging and reducing the risk of age-related diseases [28], including neurodegenerative disorders [29]. This finding is consistent with the previous results of our group in the nematode senescence phenotype assay [30].

An important area of neuron-astrocyte interactions is related to the metabolism of FAs [31], components of phospholipids in cell membranes [32], which are stored inside the cell in the form of energy-rich triglycerides located in LDs [33]. The regulation of lipid metabolism is essential to avoid the toxicity of FAs during neuronal activity and ultimately to maintain brain health [34]. Interestingly, neurons typically do not produce LDs and have a low capacity to consume FAs in mitochondria for energy production [35]. The accumulation of lipids in overactive neurons is toxic, in part, because of the sensitivity of these lipids to peroxidation. In addition, excess FAs may enter nonoxidative metabolic pathways [36], triggering excess ceramide production that is toxic to the cell, and may also disrupt mitochondrial membrane integrity [37] and dysfunction as well as reactive oxygen species (ROS) [38] membrane integrity and stimulate reactive oxygen species (ROS) production. Given the tight coordination between neurons and astrocytes, the question arises whether astrocytes help highly active neurons avoid FA toxicity by taking up excess FAs from neurons and storing them in the LD [39]. Indeed, oxidative stress in neurons triggers the formation of LDs in neighboring astrocytes, depending on the presence of ApoE [39]. This suggests that active neurons transfer their FAs to astrocytes via lipoprotein particles [8], but how this occurs and how astrocytes respond to this transfer has not been characterized. Therefore, there is a need to rescue and promote astrocyte FA oxidation during the pre-AD period for neurons to maintain normal functional metabolism.

## 2. Materials and Methods

### 2.1. Animals

APP/PSEN1-dE9 mice were purchased from Sayer (Suzhou) Biotechnology Co (Suzhou, China). The experiment was started one week after acclimatization feeding. Fifteen 4-month-old male APP/PS1 mice were randomly divided into the MOD (model) group, MOD + PHZL (low dose of PHZ) group, MOD + PHZH (high dose of PHZ) group, and seven mice of the same species and age from the same litter were selected as the blank control group, for a total of four groups of five to seven mice each. The PHZ powder was completely dissolved in dimethyl sulfoxide (DMSO) and then diluted to 5 mg/mL with distilled water, and the PHZ low and high dose groups were administered daily by gavage at doses of 50 mg/kg and 100 mg/kg, respectively. The final concentration of DMSO in the administered solution was 1%. The control and model groups were administered the same volume of distilled water by gavage for 60 days, after which behavioral tests were conducted and samples were taken. The drugs were dissolved in saline by ultrasonication. Body weights were measured daily throughout the study. Mice were housed at 22 ± 1 °C with 55 ± 5% humidity in a 12 h light-dark cycle with ad libitum access to water and food. The animal study protocol was approved by the Experimental Animal Ethics Sub-Committee of the Academic Committee of Beijing University of Chinese Medicine (protocol code: BUCM-2024121603-4223, approval date: 17 December 2024).

### 2.2. Behavioral Testing

New object recognition (NOR) was performed in an open box of 50 × 50 × 45 cm. All mice at 6 months of age were placed in the center of the box and acclimated for 10 min per day for 3 days. On the day of the test, the mouse was first placed in a box containing two identical objects for 10 min. Then, 1 h later, one of the objects was replaced with a new one, and the mouse was placed in the box again for 10 min. The animal was considered to be exploring an object when its nose tip was facing the object and the distance between the nose tip and the object was less than or equal to 2 cm. The time spent exploring the objects was recorded (nose tip toward the object ≤ 2 cm). The discrimination indices of mice in each group were statistically analyzed. The Discrimination Index (DI; DI = total time with new object/total time with both objects) [40].

The Morris Water Maze (MWM) was used to evaluate spatial learning and memory. It consisted of a circular pool (22–25 °C, 120 cm) and a platform (with a diameter of 10 cm). The pool was divided into four quadrants, and the water in the pool was of the opposite color to the mouse fur [41]. In the localization navigation experiment (days 1–4), the platform was fixed in one quadrant and hidden 1 cm below the water surface. If the platform was not founded within 60 s, the mouse was guided to the platform for another 10 s of training. Each mouse was tested once per day in each quadrant in random order. In the space exploration experiment (day 5), the platform was removed, the mice were placed in the furthest quadrant from the platform, and allowed to swim for 60 s. The video tracking system recorded each mouse’s swim route to the platform, escape latency, and the number of times it crossed the target quadrant.

### 2.3. Cell Culture

Astrocyte C8D1A cells and neuron HT22 cells were purchased from the National Experimental Cell Resource Sharing Platform. C8D1A and HT22 cells were cultured in DMEM (Concordia Cell Resource Center) containing 10% fetal bovine serum (PUMHQT-100, Curious Tech, Beijing, China) and 1% penicillin-streptomycin-gentamicin (P7630, Solarbio, Beijing, China). All cultures were maintained at 37 °C and 5% CO_2_, targeting a cell density of approximately 1 × 10^8^ cells per culture flask [42]. The second generation of cells after resuscitation was used for experiments. For cell passaging, they were diluted to 1 × 10^6^ cells/mL and inoculated in 25 T culture flasks. Astrocytes were treated by culturing in DMEM medium containing 300 nM PA for 24 h to cause disruption of their FA metabolism as a model group, and PA administration for 24 h was followed by the addition of 0.8 nM, 8 nM PHZ treatment for 24 h as a drug administration group.

### 2.4. Pharmacological Inhibition of PPARα

To investigate whether PPARα mediates the effects of PHZ on the lipid metabolism pathway in C8D1A astrocytes, we employed the PPARα-specific inhibitor GW6471. C8D1A cells were divided into the following treatment groups: (1) Control, (2) PHZ alone, (3) GW6471 alone, and (4) GW6471 + PHZ. Cells were first pre-treated with 1 μM GW6471 for 2 h to block the PPARα signaling pathway. Subsequently, the culture medium was replaced with fresh medium containing either GW6471 alone or GW6471 in combination with PHZ, and the incubation continued for 24 h. Following this treatment, the expression levels of the target protein (PPARα) and key downstream genes were detected.

### 2.5. Co-Culture Assay

The effects of astrocytes with lipid metabolism disorders on neurons were observed through the transwell experiment. In the astrocyte of the upper compartment. Lipid disorder of astrocytes in the upper compartment was caused by treatment with 300 nM PA for 24 h, and then treated with PHZ for 24 h. The lipid content and related protein expression of the neurons in the lower compartment were detected. Lipid disorder of astrocytes in the upper compartment was caused by palmitic acid (PA) treatment. We hypothesized that PHZ treatment would reverse this PA-induced lipid disorder.

### 2.6. Detection of Apoptosis and Cell Viability

Apoptosis was examined using the Annexin V-PI double staining kit (CA1020, Solarbio). Adherent and floating cells were collected 24 h after PA modeling and 24 h after PHZ administration, stained with Annexin V and PI according to the manufacturer’s instructions, and analyzed using flow cytometry.

Cell viability was assayed by a CCK-8 assay kit (CA1210, Solarbio). Cells were inoculated in 96-well plates at a density of 10^4^ cells and treated as described above. At the end of PHZ administration, 10 μL of CCK-8 was added to each well and incubated for 30 min. The absorbance of each well was measured at 450 nm using an enzyme meter.

### 2.7. Mitochondrial Membrane Potential Assay

C8D1A cells were inoculated in 6-well plates and incubated at 37 °C for 24 h. Cells were treated with drugs for 24 h (NC group: complete medium; PA group: 300 nM PA; PHZ group: 300 nM PA + 0.8 nM PHZ). Each group had 3 replicate wells, and each well contained 2 mL of culture medium. After discarding the medium, cells were washed once with PBS, and then 1 mL of cell culture medium and 1 mL of JC-1 staining working solution (M8650, Solarbio) were added. The mixture was thoroughly mixed and incubated in a cell culture incubator at 37 °C for 20 min. After the incubation period, the supernatant was removed and the cells were washed twice with JC-1 Staining Buffer (1×). Subsequently, 2 mL of cell culture medium was added and observed under a fluorescence microscope [43] (RVL-100, ECHO, San Diego, CA, USA), and the fluorescence intensity was measured. Mean values were analyzed by ImageJ 1.8.

### 2.8. Quantitative Analysis of NEFA and TG

The free fatty acid (NEFA) kit (A042-2-1, Nanjing Construction Bioengineering Institute, Nanjing, China) was used for the experiments. Take the supernatant of each group of cells, after adding reagent I, incubate at 37 °C for 5 min, read the absorbance value A1 at 546 nm, then add reagent II, incubate at 37 °C for 5 min, read the absorbance value A2, and calculate the △A = A2 − A1.

Triglyceride (TG) kit (A110-1-1, Nanjing Jianjian Bioengineering Institute, Nanjing, China) was used. The cell suspension was centrifuged at 1000 r for 10 min. After discarding the supernatant, the cell pellet was washed one to two times with an isotonic phosphate buffer (0.1 mol/L, pH 7.0–7.4). Following each wash, the cells were centrifuged again at 1000 r for 10 min and the supernatant was discarded. The final cell pellet was retained for subsequent analysis. Then, the cells were lysed with lysis buffer (TritonX-100, 1–2%) for 30 min. The lysate was centrifuged at 12,000 r for 10 min to remove insoluble debris. The resulting supernatant was then collected, and its absorbance was measured at 500 nm using a microplate reader [44].

Triglycerides and free fatty acids were analyzed by the Triglyceride and Free Fatty Acid Quantification Kit according to the manufacturer’s instructions. Three independent replicates were performed for each group.

### 2.9. Nile Red Staining

Nile red staining experiments were performed using the Lipid Fluorescence Staining Kit to measure lipid accumulation in astrocytes according to the instructions of the kit (G1264, Solarbio). Cells were fixed with lipid fixative and then incubated with the appropriate volume of staining solution for 20 min away from light. Fluorescence images from three independent samples were captured using an imaging system (RVL-100, ECHO) under a 485 nm excitation light source and analyzed by ImageJ to obtain mean values of Nile Red positive fluorescence intensity under a uniform scale field of view.

### 2.10. Western Blotting

Protein solutions from each group of 5–7 mice were denatured with 5 × sample buffer at 100 °C for 10 min and stored at −20 °C. The protein solutions were then analyzed by Bio-Rad Protein Assay. Samples of 20 to 40 μg of total protein were separated by SDS-polyacrylamide gel electrophoresis using the Bio-Rad Protein Assay and transferred to a polyvinylidene difluoride (PVDF) membrane. The PVDF membrane was incubated at room temperature in 5% skimmed milk powder for 2 h and then incubated overnight with the primary antibody (Table 1) at 4 °C. The corresponding secondary antibodies were incubated at room temperature for 1 h. The bands were displayed using ECL hypersensitive luminescence solution (E1060, Lablead, Beijing, China), and the gray values were analyzed using ImageJ and normalized to the relative protein level.

### 2.11. Quantitative Real-Time Reverse Transcription PCR (qRT-PCR)

Total RNA was extracted with TRIzol (15596026, Invitrogen, Waltham, MA, USA). cDNA was synthesized using the PrimeScript RT reagent Kit (RR037A, Takara, Beijing, China) in a reaction of 1 μg of total RNA per reaction. Relative mRNA levels were quantified by qRT–PCR using the SYBR^®^ Green Premix Pro Taq HS qPCR Kit (Q712, Accurate Biology, Hunan, China) with the following specific primers [45]. The primer sequences for qPCR are listed in Table 2. The mRNA expression levels were normalized to those of GAPDH.

### 2.12. Immunofluorescence of Aβ

Whole brains were isolated and post-fixed by soaking in 4% PFA overnight at 4 °C, then transferred to 30% sucrose (S8271, Solarbio) for dehydration for 3–5 days. Hemi-brain sections were obtained using a pathology slicer (RM2016, Leica, Wetzlar, Germany). Brain sections were closed with 10% goat serum (SL038, Solarbio) for 1–2 h at room temperature, and then incubated with Aβ primary antibody (71-5800, Thermo Fisher, Waltham, MA, USA) at 4 °C overnight. The sections were washed with PBS (P1020-5L, Solarbio) and then incubated with HRP-labeled goat anti-rabbit secondary antibody (5220-0336, SeraCare, Beijing, China). Then, the brain sections were blocked on slides and dried in the dark, and the tissues were incubated with drops of tyramine salt fluorescein CY3 for 20 min at room temperature. Drops of DAPI staining solution were added to the circles and incubated for 10 min at room temperature, protected from light. The slides were placed in PBS (pH 7.4) in a destaining shaker and washed by shaking 3 times for 5 min each time. The sections were shaken slightly to dry and then blocked with an anti-fluorescence-quenching blocking agent [46]. For each animal, multiple non-overlapping fields of view were systematically captured from the hippocampal CA1 and DG regions and the prefrontal cortex to ensure comprehensive sampling. Sections were observed under a fluorescence microscope, and images were captured and analyzed by ImageJ software for the proportion of area occupied by Aβ in the tissue sections, Aβ Area%.

### 2.13. Oil Red O Staining Co-Labeled with GFAP Immunofluorescence

The frozen slices of liver tissue and hepatocytes were rinsed slightly with distilled water and soaked in 60% isopropanol for 20 s before being stained with Oil red O solution (G1261, Solarbio) for 30 min at room temperature. The slides were washed slightly in 60% isopropanol to remove the dye solution, followed by rinsing with distilled water [47].

After staining the lipid plates, place them in TBS for the first wash for 1 to 3 min. Wash with TBS for another 10 min three times. Add 5–10%BSA and block for 10–30 min. Then directly add the primary antibody GFAP (16825-1-AP, Proteintech, Hubei, China) and store in a 4 °C refrigerator overnight. The next day, wash with TBS for the first time for 1 to 3 min, and do this 3 times every 10 min. Incubate at room temperature in the dark with the Goat Anti-Rabbit IgG (Y1048, lablead, Beijing, China) for two hours. After the incubation, wash with TBS and then with pure water as usual. Let it dry and then seal with a mounting medium containing DAPI. Images were captured with a fluorescence scanner (OLYMPUS, Tokyo, Japan).

### 2.14. Molecular Docking and Interactions

The crystal structure of the PPARα protein used for docking was obtained by downloading from the PDB database with PDB ID 3VI8 for PPARα, and the 3D structure of the small molecule phlorizin was obtained by downloading from the PUBCHEM database and energy minimized under the MMFF94 force field. In this study, molecular docking work was performed using AutoDock Vina 1.2.3 software [48]. Prior to the start of docking, the receptor proteins were processed using PyMol 2.5.5 [49], which included the removal of water molecules, salt ions, and small molecules. Subsequently, the docking box was set up with the crystal ligand center in the center of the box, and the size of the box was 30 × 30 × 30 cubic angstroms. In addition, all processed small molecules as well as receptor proteins were converted to the PDBQT format necessary for AutoDock Vina 1.2.3 docking using ADFR suite 1.03 [50]. For docking, the exhaustiveness of the global search was set to 32, and the rest of the parameters were left at their default settings. The output docked conformations with the highest scores were considered by us as binding conformations, and finally, the docking results were visualized and analyzed using PyMol 2.5.5.

### 2.15. Surface Plasmon Resonance (SPR)

SPR was performed according to the manufacturer’s instructions. The CM5 sensor chip (29149603, Cytiva, Logan, UT, USA) was initially activated with 0.4 M 1-ethyl-3-(3-dimethylaminopropyl) carbodiimide hydrochloride and 10 mM N-hydroxysuccinimide at a flow rate of 5 μL/min for 20 min. The CM5 sensor chip (29149603, Cytiva) was activated with 1 mL sodium acetate (10 mM, pH 4.5) and then flowed over the activated surface to reach the target response value (RU). The remaining activation sites on the chip were blocked with 45 μL ethanolamine (1 M, pH 8.5). Real-time assays were recorded using a Biacore X100 instrument (Cytiva, Logan, UT, USA) at a flow rate of 30 μL/min. Subsequently, to determine the equilibrium dissociation constant (KD) of PPARα with PHZ, serially diluted PHZ was applied in 150 mM phosphate-buffered saline (pH 7.4), and interactions with PPARα and PHZ immobilized on the surface of the chip were analyzed using the BIA assessment procedure.

### 2.16. Detection of ACADs Enzyme Activity

Mouse brain tissues were weighed and homogenized on ice in nine volumes of pre-cooled RIPA lysis buffer containing 1% protease inhibitor cocktail. The homogenates were centrifuged at 12,000× *g* for 20 min at 4 °C, and the supernatants were collected. The total protein concentration was determined using the BCA protein assay kit (P1511, APPLYGEN, Beijing, China). All samples were diluted to a uniform concentration prior to the ELISA. The concentration of ACADs was determined using a commercial mouse ELISA kit according to the manufacturer’s instructions (JM-13703M1, JM-13786M1, JM-13791M1, JM-13797M1, JINEMEI BIOTECHNOLOGY, Shandong, China). A standard curve was generated by plotting the mean absorbance for each standard concentration against its known value. The concentration of ACADs in unknown samples was interpolated from the standard curve using a four-parameter logistic (4-PL) curve fit.

### 2.17. Statistical Analysis

All statistical analyses in this study were performed using GraphPad Prism software, version 9.0. The normality of continuous data was assessed using the Shapiro–Wilk test, and homogeneity of variances was verified by the Brown-Forsythe test. All data met the assumptions for one-way ANOVA. Whenever the one-way ANOVA revealed a statistically significant overall difference (*p* < 0.05), we proceeded with post hoc pairwise comparisons using Tukey’s Honest Significant Difference (HSD) test. All statistical tests were two-sided, and a *p*-value of less than 0.05 was considered statistically significant. Data are presented as mean ± standard deviation.

## 3. Results

### 3.1. PHZ Alleviated Cognitive Dysfunction in APP/PS1 Mice

**Figure 1:**
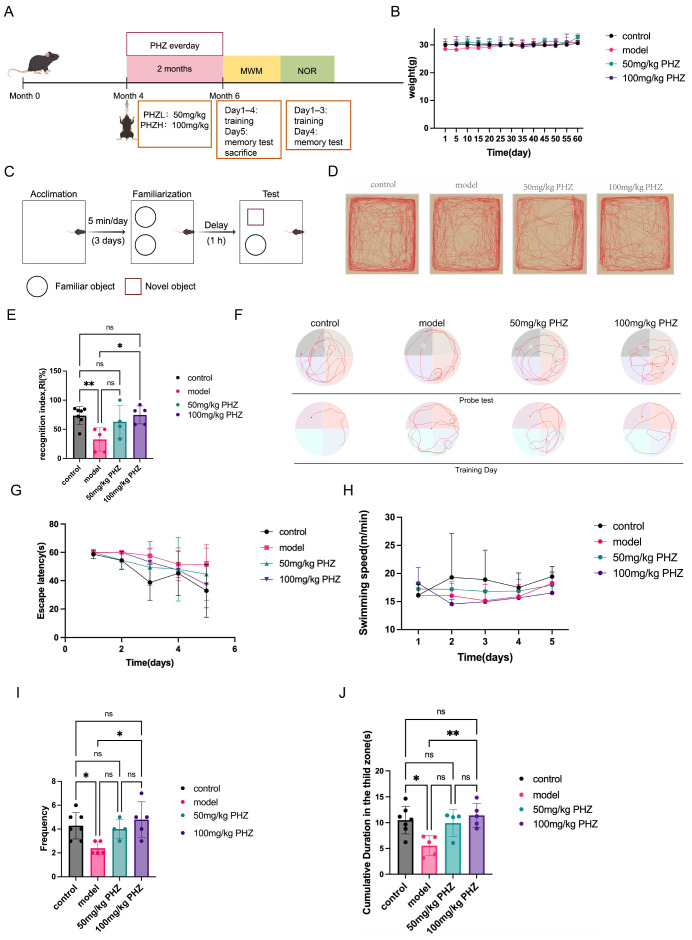
Oral administration of PHZ significantly improved the spatial memory ability of APP/PS1 mice by promoting astrocyte fatty acid metabolism per group (*n* = 4−7, * *p* < 0.05, ** *p* < 0.01, one−way ANOVA). (**A**) Schematic diagram of APP/PS1 mice administered PHZ by gavage and behavioral tests. (**B**) Changes in body weights of APP/PS1 mice in each group during gavage administration. (**C**) Experimental details and operational procedures in the new object recognition (NOR) test for 6−month−old APP/PS1 mice. (**D**) Mouse trajectories from each group tracking the NOR (New: new Object, Old: old Object). (**E**) New object recognition index. (*n* = 4−7, ns *p* ≥ 0.05, * *p* < 0.05, ** *p* < 0.01, one−way ANOVA) (**F**–**J**) Morris water maze test to assess the effect of dietary PHZ on spatial memory in 6−month−old APP/PS1 mice. Representative insets show the trajectory of the maze on training day 4 and the final probe test for each experimental group (**F**). On the training day, escape latency (time for mice to find the platform) (**H**) and swimming speed (**G**) were measured. In the final probe test, the frequency of stay in the target quadrant where the platform was located in each group of mice (**I**) and the path across the target quadrant (**J**) were assessed (*n* = 4−7, ns *p* ≥ 0.05, * *p* < 0.05, ** *p* < 0.01, one−way ANOVA).

To investigate the effect of PHZ on cognitive dysfunction in APP/PS1 mice, we administered PHZ for two months and analyzed cognitive behaviors using NOR and MWM recordings. Two months of PHZ administration had no effect on body weight (Figure 1B), while significantly altering cognitive behaviors (Figure 1C–J). The novel object recognition test revealed a significant decrease in the recognition index (RI) of APP/PS1 mice compared to control animals. PHZ treatment effectively attenuated this cognitive deficit in a dose-dependent manner (Figure 1C–E). Specifically, the average RI increased by more than 90% in the 50 mg/kg PHZ group and by nearly 100% in the 100 mg/kg PHZ group. These results indicate that PHZ plays a beneficial role in alleviating short-term memory impairment in AD model mice. Furthermore, we assessed spatial memory using a water maze experiment. Although neither escape latency nor swimming speed was altered, both frequency and cumulative duration in the target zone were reduced in APP/PS1 mice (Figure 1F–J). In APP/PS1 mice, PHZ administration increased the target quadrant frequency and cumulative time of mice by about 100% (Figure 1F–J), and there was no difference between the 100 mg/kg PHZ group and the control group, suggesting that the spatial memory ability of mice after high-dose PHZ administration was no longer significantly different from that of normal mice. These behavioral results suggest that PHZ alleviates multiple memory impairments in AD mice.

### 3.2. PHZ Suppressed the Production of Aβ in Hippocampus and Cortex of APP/PS1 Mice

**Figure 2:**
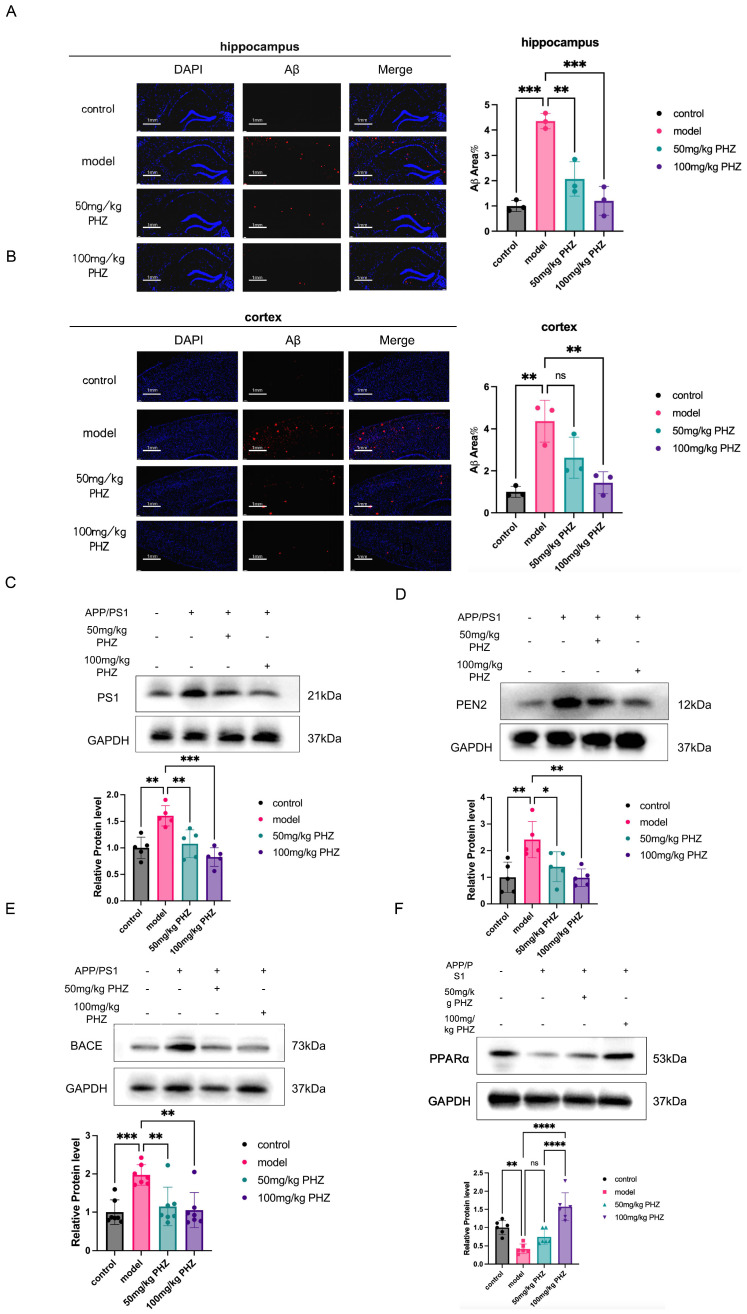
(**A**) Expression of Aβ in the hippocampus, DAPI was marked in blue and Aβ in red. (*n* = 3, ** *p* < 0.01, *** *p* < 0.001, one-way ANOVA). (**B**) Expression of Aβ in the prefrontal cortex (*n* = 3, ns *p* ≥ 0.05, ** *p* < 0.01, one-way ANOVA). (**C**) PS1 protein expression in mice in each group, and “+” means that the animals are APP/PS1 mice or that the animals in this group have PHZ administration; “−” means that the animals were not APP/PS1 mice (blank mice) or that the group was not administered PHZ. (*n* = 5, ** *p* < 0.01, *** *p* < 0.001, one-way ANOVA). (**D**) PEN2 protein expression in each group (*n* = 5, * *p* < 0.05, ** *p* < 0.01, one-way ANOVA). (**E**) BACE protein expression in mice in each group (*n* = 7, ns *p* ≥ 0.05, ** *p* < 0.01, *** *p* < 0.001, one-way ANOVA). (**F**) PPARα protein expression in APP/PS1 mice in each group (*n* = 6, ns *p* ≥ 0.05, ** *p* < 0.01, **** *p* < 0.0001, one-way ANOVA).

Memories are formed in the hippocampus and stored in the cortex. Using immunostaining, we investigated the effect of PHZ on Aβ production, a pathological marker of AD, in both hippocampus and cortex (Figure 2A,B). Aβ deposition was observed in both the hippocampus and prefrontal cortex of APP/PS1 mice at 6 months of age, which was reduced by PHZ intervention. 50 mg/kg PHZ reduced Aβ deposition by about 50%, and 100 mg/kg PHZ reduced pathological markers by nearly 75%. We further quantified the protein expression of the Aβ synthetase subunit. Protein levels of β-secretase subunit BACE1, γ-secretase subunit PS1, and γ-secretase subunit PEN2 were increased in the brain of APP/PS1 mice. PHZ treatment downregulated these synthetase enzymes (Figure 2C–E), suggesting that PHZ reduces neuronal pathological marker content by reducing Aβ production. As a key upstream of fatty acid catabolism, PPARα is involved in the pathological process of AD. Therefore, we investigated the effect of PHZ on central lipid metabolism. We examined the protein expression of PPARα in the brain tissue of APP/PS1 mice and found that its expression was decreased. PHZ administration for 2 months significantly increased PPARα levels, with a nearly 300% increase in the 100 mg/kg PHZ group (Figure 2F).

### 3.3. PHZ Ameliorated Lipid Metabolism Disorders in Astrocytes

**Figure 3:**
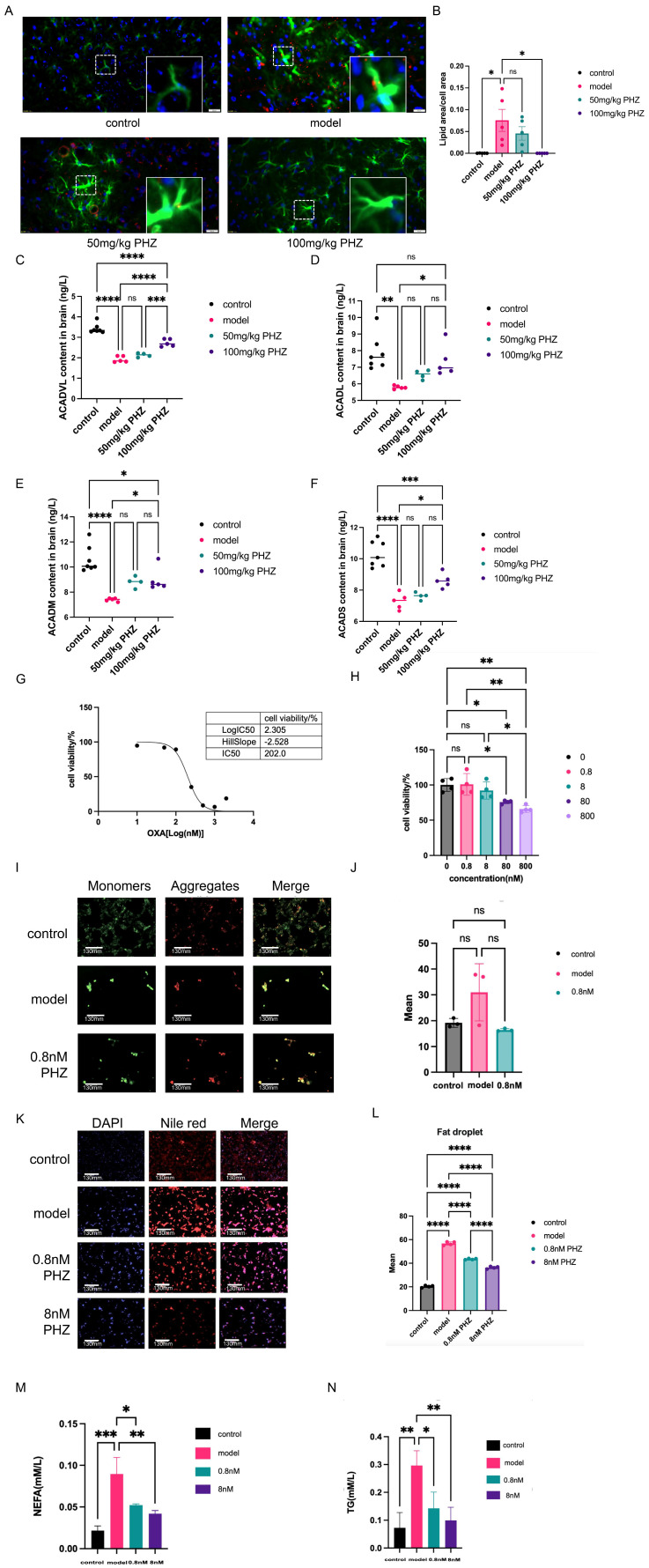
(**A**) GFAP and oil red O staining in APP/PS1 mice in each group. Lipids are labeled in red and astrocytes in green, and the labeling results within the white border of each group are magnified in the lower right corner. (**B**) Lipid area/cell area in each group (*n* = 5, ns *p* ≥ 0.05, * *p* < 0.05, one-way ANOVA). (**C**) The enzyme activity of ACADVL was detected by Elisa (*n* = 4–7, ns *p* ≥ 0.05, *** *p* < 0.001, **** *p* < 0.0001, one-way ANOVA). (**D**) The enzyme activity of ACADL was detected by Elisa (*n* = 4–7, ns *p* ≥ 0.05, * *p* < 0.05, ** *p* < 0.01, one-way ANOVA). (**E**) The enzyme activity of ACADM was detected by Elisa (*n* = 4.7, ns *p* ≥ 0.05, * *p* < 0.05, **** *p* < 0.0001, one-way ANOVA). (**F**) The enzyme activity of ACADS was detected by Elisa (*n* = 4–7, ns *p* ≥ 0.05, * *p* < 0.05, *** *p* < 0.001, **** *p* < 0.0001, one-way ANOVA). (**G**) Effects of different concentrations of PA on astrocyte activity. (**H**) Effects of different concentrations of PHZ on astrocyte activity (*n* = 3–4, ns *p* ≥ 0.05, * *p* < 0.05, ** *p* < 0.01, one-way ANOVA). (**I**) Modeling of disorders of lipid metabolism in astrocytes and assay of mitochondrial membrane potential after administration of PHZ. (**J**) Intensity analysis of mitochondrial membrane potential fluorograms (*n* = 3, ns *p* ≥ 0.05, one-way ANOVA). (**K**) Nile red staining of intracellular macromolecular lipid droplets in astrocytes of each group. (**L**) Effect of PHZ on the content of lipid droplets in astrocytes with disorders of lipid metabolism (*n* = 4, **** *p* < 0.0001, one-way ANOVA). (**M**) Extracellular free fatty acid content of astrocytes before and after drug administration (*n* = 3, * *p* < 0.05, ** *p* < 0.01, *** *p* < 0.001, one-way ANOVA). (**N**) Intracellular TG content of astrocytes before and after drug administration (*n* = 4, * *p* < 0.05, ** *p* < 0.01, one-way ANOVA).

PPARα, a key regulator of lipid metabolism, can be influenced by signals originating from the central nervous system (CNS). To further investigate the improvement of lipid metabolism in astrocytes of APP/PS1 mice by PHZ, we co-labeled Oil Red O and GFAP, and we found that lipid expression in astrocytes of APP/PS1 mice increased, while it decreased after PHZ administration (Figure 3A,B). Given the central role of mitochondrial fatty acid β-oxidation (FAO) in brain energy homeostasis and its dysfunction in Alzheimer’s disease (AD), we sought to directly assess the functional capacity of this pathway by measuring the enzymatic content of key Acyl-CoA Dehydrogenases (ACADs). Notably, we observed a significant reduction in ACADs content in AD model brain tissues. Our intervention ameliorated this deficit, restoring ACADs content to more than 20% above the baseline level in the AD model group (Figure 3C–F). Astrocytes, as key sites of central lipid metabolism, maintain their normal functions is of great significance. Abnormal degradation of fatty acids leads to lipid droplet accumulation, which is a characteristic of AD. Then observe the effect of PHZ on lipid metabolism disorders. The lipid droplets of C8D1A in astrocytes treated with PA increased, while PHZ reduced them (Figure 3G,H). In addition, the contents of extracellular free fatty acids and intracellular TG in astrocytes were both increased in C8D1A cells with PA, suggesting that lipid metabolism disorders not only lead to the accumulation of intracellular macromolecular lipids but also the increase in extracellular free fatty acids, indicating that the ability of astrocytes to accept lipids in the environment is also reduced. PHZ inhibited the production of free fatty acids and triglycerides in a dose-dependent manner (Figure 3I,J), indicating that PHZ can promote intracellular lipolysis to decrease free fatty acids, and thus avoid lipotoxicity generation by brain lipid accumulation. Mitochondrial membrane potential, an early event of apoptosis, was not altered after PA application, as well as with PHZ treatment (Figure 3E,F). These results demonstrated that the disorder of lipid metabolism is an early pathological change that occurs before apoptosis in astrocytes. PHZ intervention may have contributed to preventing the occurrence and development of AD through repairing these early pathological changes.

### 3.4. PPARα Played as a Key Target of PHZ to Regulate Fatty Acid Catabolism

**Figure 4:**
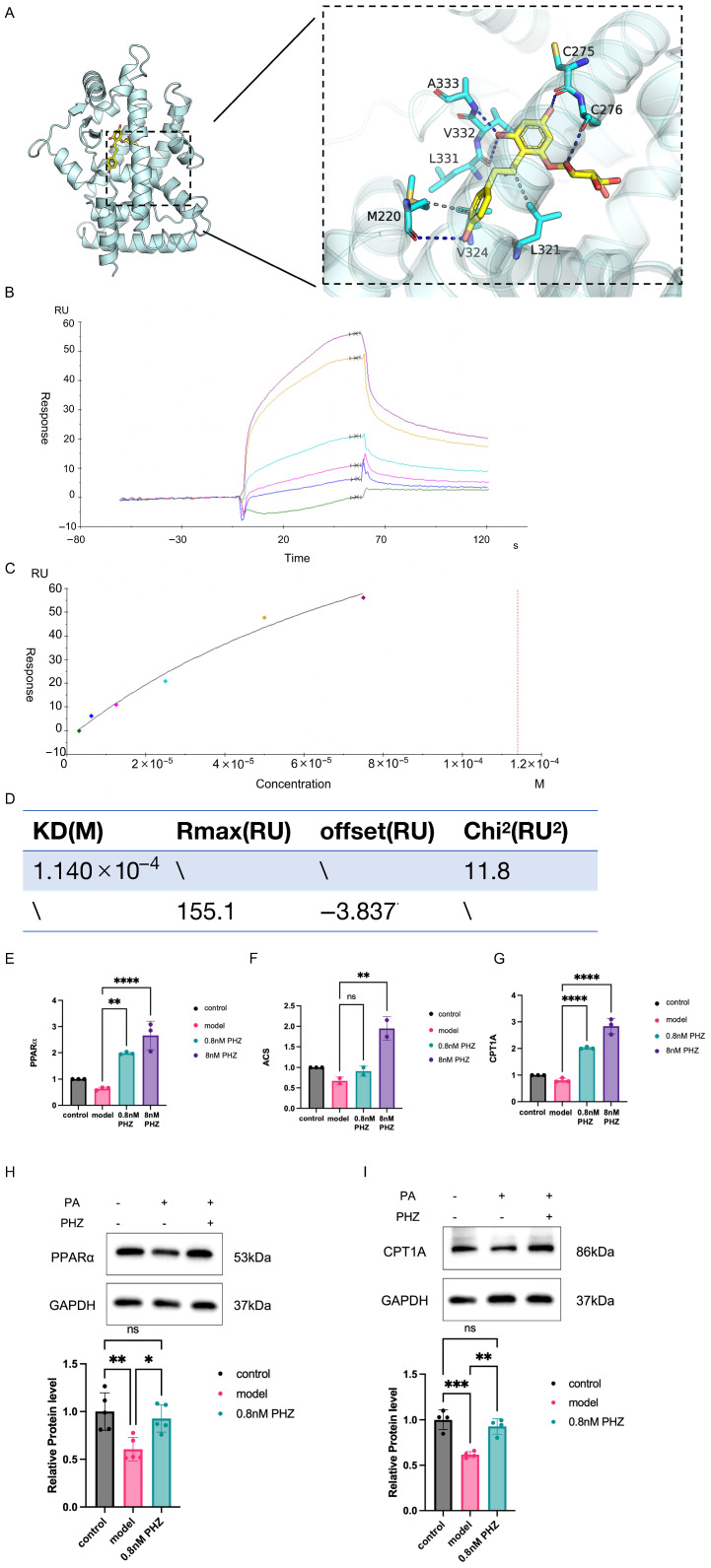
(**A**) Based on the binding pattern of PPARα and phlorizin obtained by docking, the left figure shows the overall view and the right figure shows the partial view, in which the yellow stick is the small molecule, the cyan cartoon is the protein, the blue line indicates hydrogen bonding interaction, and the gray dotted line indicates hydrophobic interaction. (**B**) Docked sensing plots of PPARα and 3.125–75 PHZ molecules, each curve represents the sensing signal at different concentrations of PHZ, that is, the sensing map of PHZ and PPARα. (**C**) Docked fitting plots of PPARα and PHZ molecules, the colored dots are the response values of PHZ and PPARα at different concentrations, and the curve is the fitting plot of PHZ and PPARα at different concentrations. (**D**) Data related to sensing and fitting plots. (**E**) PPARα mRNA content of astrocytes in each group (*n* = 3, ** *p* < 0.01, **** *p* < 0.0001, one-way ANOVA). (**F**) ACS mRNA content of astrocytes in each group (*n* = 3, ns *p* ≥ 0.05, ** *p* < 0.01, one-way ANOVA). (**G**) CPT1A mRNA content of astrocytes in each group (*n* = 3, **** *p* < 0.0001, one-way ANOVA). (**H**) PPARα protein expression in astrocyte, and “+” means that cells have been treated with PA or PHZ, and “−” means that they have not been treated with PA or PHZ. (*n* = 5, ns *p* ≥ 0.05, * *p* < 0.05, ** *p* < 0.01, one-way ANOVA). (**I**) CPT1A protein expression in astrocyte (*n* = 4, ns *p* ≥ 0.05, ** *p* < 0.01, *** *p* < 0.001, one-way ANOVA).

The main bearers of fatty acid metabolism in the brain are astrocytes. We detected PPARα expression using an in vitro model with the disorder of FA metabolism. By quantification of mRNA and protein content, we found that PA application induced a decrease in both mRNA and protein levels of PPARα in C8D1A cells, which were promoted by PHZ (Figure 4E,H), suggesting that astrocytes may be an important site for PHZ to exert its drug effects. Then we determined that PHZ could well bind to PPARα using molecular docking and SPR techniques (Figure 4A–D). In the molecular docking results, global views are shown on the left and local views on the right, yellow bars are small molecules, cyan are proteins, blue lines represent hydrogen bond interactions, and gray dashed lines represent hydrophobic interactions. The figure shows that phloridzin forms hydrogen bonds with LEU-331, MET-220, CYS-276, CYS-275, and ALA-333 on the PPARα protein. The formation of hydrogen bonds leads to closer binding between proteins and small molecules. Hydrophobic interactions with LEU-321, MET-220, VAL-332, and VAL-324 provide strong van der Waals forces (Figure 4A). We then examined the binding of PHZ to PPAR by SPR experiments with a dissociation constant of 1.04 × 10^−4^ (Figure 4B–D).

We further detected two downstream of PPARα, ACS and CPT1A. Although neither ACS nor CPT1A mRNAs were altered, a decreased CPT1A protein level was observed in C8D1A cells with PA application (Figure 4I). PHZ showed a strong effect in increasing the expression levels of these two downstream of PPARα (Figure 4F,G). Above all, these results suggested that PHZ could upregulate the PPARα pathway in fatty acid catabolism in astrocytes.

**Figure 5:**
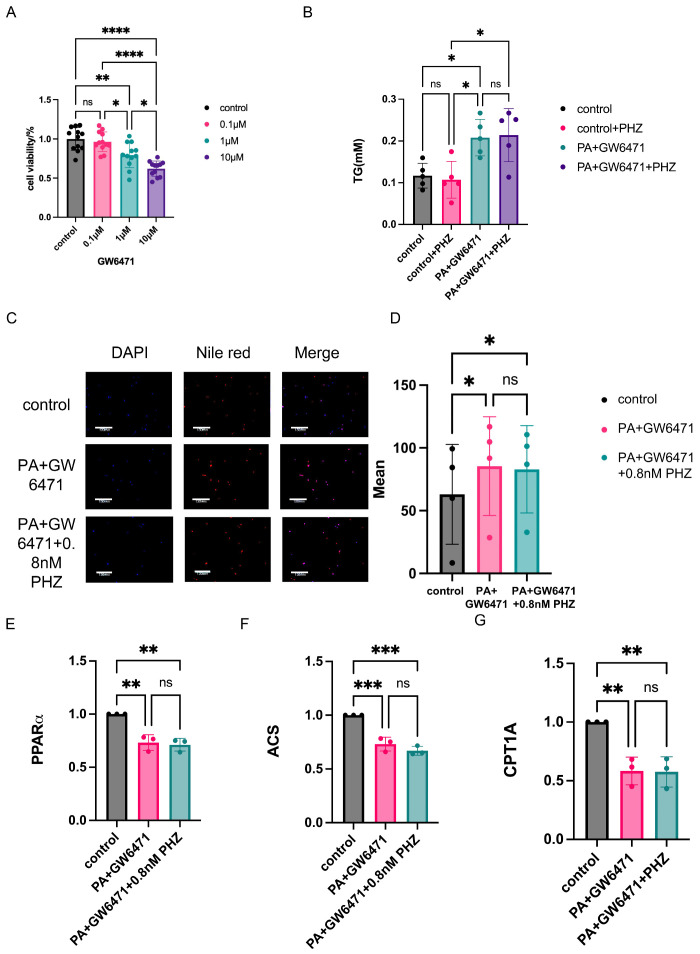
(**A**) Effect of different concentrations of GW6471 on astrocyte activity (*n* = 10–12, ns *p* ≥ 0.05, * *p* < 0.05, ** *p* < 0.01, **** *p* < 0.0001, one-way ANOVA). (**B**) Effect of the PPARα inhibitor GW6471 on intracellular TG content in astrocytes (*n* = 5, ns *p* ≥ 0.05, * *p* < 0.05, one-way ANOVA). (**C**) Nile red staining of intracellular macromolecular lipid droplets in astrocytes of each group. (**D**) Effect of GW6471 on the content of lipid droplets in astrocytes with disorders of lipid metabolism (*n* = 4, ns *p* ≥ 0.05, * *p* < 0.05, one-way ANOVA). (**E**) Effect of PHZ on astrocyte PPARα mRNA by addition of GW6471 glial cell PPARα mRNA (*n* = 3, ns *p* ≥ 0.05, ** *p* < 0.01, one-way ANOVA). (**F**) Effect of PHZ on astrocyte ACS mRNA after addition of GW6471 (*n* = 3, ns *p* ≥ 0.05, *** *p* < 0.001, one-way ANOVA). (**G**) Effect of PHZ on astrocyte CPT1A mRNA after addition of GW6471 (*n* = 3, ns *p* ≥ 0.05, ** *p* < 0.01, one-way ANOVA).

In order to further investigate the role of PHZ through PPARα, we added the PPARα inhibitor GW6471 (Figure 5A) and found that after the addition of GW6471, the intracellular TG and macromolecular lipid droplets were not reduced (Figure 5B–D), and the fatty acid catabolic pathway could not be activated. The effect of PHZ was inhibited by GW6471 (Figure 5E–G), suggesting that PHZ regulates astrocyte fatty acid disorders through PPARα.

### 3.5. PHZ Regulated Lipid Metabolism in Astrocytes to Reduce Neuronal Lipotoxicity and Aβ Production

**Figure 6:**
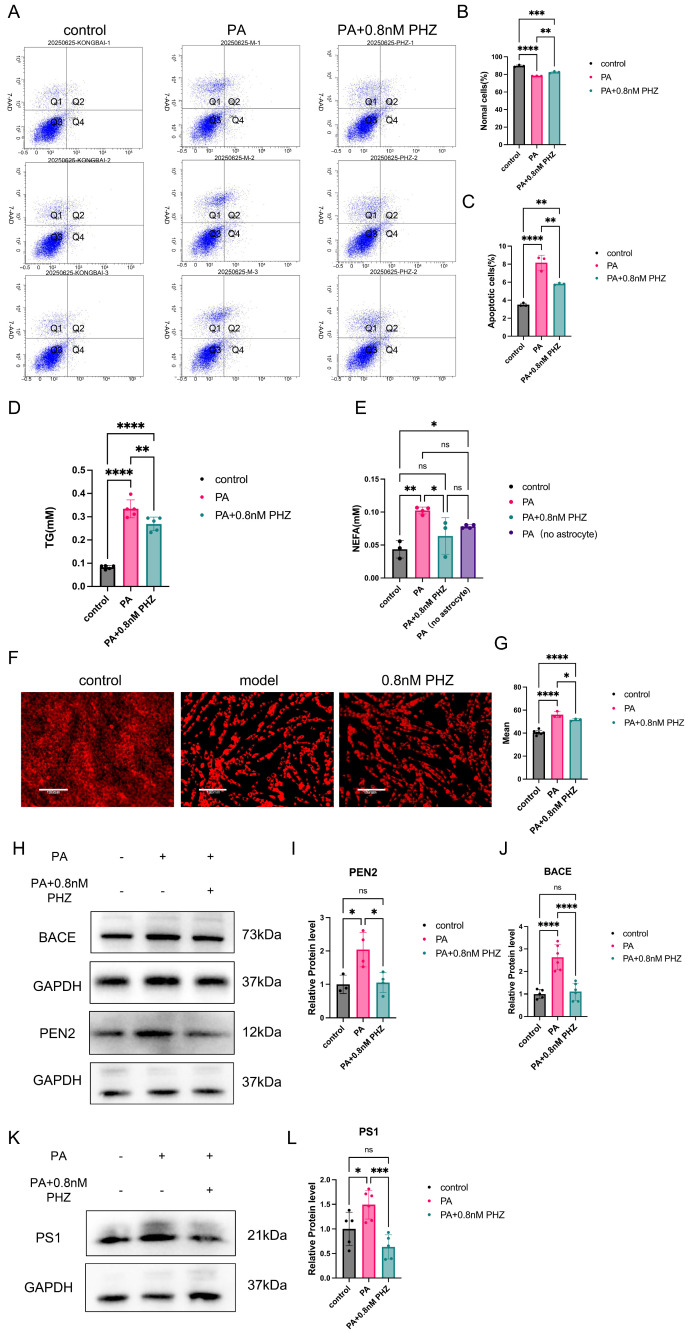
(**A**) Neuronal flow apoptosis in each group, and blue marks cells that enter different circles. (*n* = 4, ns *p* ≥ 0.05, **** *p* < 0.0001, one-way ANOVA). (**B**) The proportion of normal living cells in each group (*n* = 3, ** *p* < 0.01, *** *p* < 0.001, **** *p* < 0.0001, one-way ANOVA). (**C**) Proportion of apoptotic cells in each group (*n* = 3, ** *p* < 0.01, **** *p* < 0.0001, one-way ANOVA). (**D**) Intracellular TG content in neurons of each group (*n* = 5, ns *p* ≥ 0.05, ** *p* < 0.01, **** *p* < 0.0001, one-way ANOVA). (**E**) Content of extracellular free fatty acids in each group (*n* = 3–4, ns *p* ≥ 0.05, * *p* < 0.05, ** *p* < 0.01, one-way ANOVA). (**F**,**G**) Neuronal intracellular lipid droplet Nile red staining (Lipid droplets accumulated in the cells are marked in red). and fluorescence intensity in each group (*n* = 3–4, * *p* < 0.05, **** *p* < 0.0001, one-way ANOVA). (**H**–**L**) Protein expression of γ-secretase and β-secretase subunits in co-cultured neurons (“+” means that cells have been treated with PA or PHZ, and “−” means that they have not been treated with PA or PHZ.). (**I**) Protein expression of PEN2 in co-cultured neurons (*n* = 3–4 ns *p* ≥ 0.05, * *p* < 0.05, one-way ANOVA). (**J**) Protein expression of BACE in co-cultured neurons (*n* = 5–6, ns *p* ≥ 0.05, **** *p* < 0.0001, one-way ANOVA). (**L**) Protein expression of PS1 in co-cultured neurons (*n* = 5, ns *p* ≥ 0.05, * *p* < 0.05, *** *p* < 0.001, one-way ANOVA).

To study the impact of astrocyte lipid metabolism disorders on neurons, we co-cultured astrocytes with neurons. Through flow cytometry apoptosis, we found that lipid metabolism disorders in astrocytes have an impact on neuronal activity (Figure 6A). Astrocytes with lipid metabolism disorders lead to a decrease in the proportion of normal neurons (Figure 6B) and an increase in the proportion of apoptotic cells (Figure 6C). We found that lipid metabolism disorder in astrocytes leads to an increase in extracellular free fatty acids in neurons. At the same time, the content of macromolecular lipid droplets and TG in neurons increases (Figure 6D,F,G), indicating that lipid metabolism disorder in astrocytes may affect intracellular lipid accumulation in neurons by increasing lipids in the environment where neurons are located. We found that lipid accumulation promoted the expression of Aβ synthetase (Figure 6D–F) and that the expression of synthetase subunits decreased by approximately 50% after PHZ treatment, suggesting that PHZ could maintain normal neuronal function by improving abnormal lipid metabolism in astrocytes, reducing neuronal lipotoxicity, and avoiding AD marker production. While this observation in the co-culture system is consistent with the transfer of fatty acids from neurons to astrocytes, it does not rule out other indirect mechanisms.

## 4. Discussion

In recent years, many academic studies have discovered that lipid metabolism abnormalities are closely associated with Alzheimer’s disease (AD) [51]. AD patients often exhibit central lipid metabolism disorders [3], which frequently precede cognitive impairment and exacerbate the onset and progression of AD [52]. Central lipid metabolism disorders represent a key early driver of Alzheimer’s disease. Astrocytes, as key regulators of central lipid metabolism, form the core of fatty acid metabolism in the brain [53]. Our experimental findings reveal increased lipid content in the brains of six-month-old AD model mice, with significantly elevated lipid accumulation specifically within astrocytes, indicating abnormal lipid metabolism in these cells. In addition, we observed significant reductions in the levels of key enzymes of ACADs in the brains of AD model mice, which directly confirmed the severe dysfunction of mitochondrial fatty acid metabolism in the AD brain. PHZ intervention can significantly increase the content of ACADs, reverse the metabolic abnormalities downstream of the FAO pathway, and reduce lipid accumulation in AD model mice. Impaired learning and memory functions are common symptoms in AD patients [54]. We observed markedly impaired learning and memory abilities in AD model mice, whereas PHZ-treated mice exhibited restored cognitive function. As a key pathological biomarker of AD [55], Aβ staining in mouse brain tissue revealed accumulation in the hippocampus and cortex of AD models. PHZ treatment improved Aβ levels with a dose-dependent reduction. Aβ is generated by cleavage of the amyloid precursor protein (APP) via β- and γ-secretases [56], whose expression promotes Aβ production [57]. Further detection of β- and γ-secretase subunits (BACE, PS1, and PEN2) revealed increased expression of Aβ-synthesizing enzymes in AD model mice, while the PHZ group exhibited decreased expression of these enzyme subunits.

Central lipid accumulation is closely associated with astrocytic lipid metabolism [5]. As the most active site for fatty acid catabolism in the central nervous system [58], astrocytes play a crucial role in maintaining lipid homeostasis within the brain [59]. To investigate the molecular mechanism by which PHZ improves central lipid metabolism disorders, we selected astrocytes with disrupted lipid metabolism for in vitro validation. PA is a commonly used agent for modeling lipid metabolism disorders [60]. Our lipid droplet staining results revealed that PA significantly increased intracellular lipid droplets and triglycerides in astrocytes, while also markedly elevating extracellular free fatty acid levels. This suggests a potential reduction in astrocytes’ capacity to take up lipids from the environment. These lipid alterations were significantly suppressed following PHZ treatment. Previous studies by our group revealed that PHZ possesses multiple bioactivities, including anti-aging effects, and can activate the NHR-49 gene homologous to PPARα in C. elegans [24]. This activation regulates lipid metabolism in nematodes, extends their lifespan, and improves pathological phenotypes of AD [30]. Our findings further demonstrate that PHZ binds well to PPARα in vitro with a low dissociation constant. In vitro experiments show that PHZ can upregulate PPARα expression in astrocytes with lipid metabolism disorders, as well as the downstream CPT1 and ACS gene and protein expression. Following treatment with the PPARα inhibitor GW6471, PHZ’s ability to improve astrocytic lipid metabolism was abolished, and PHZ failed to promote increased PPARα/CPT1/ACS expression. These findings confirm that PHZ ameliorates astrocytic lipid metabolism disorders by enhancing PPARα expression.

Increasing research indicates that lipid accumulation within neurons exhibits toxicity [61]. This toxicity arises not only from the lipids’ sensitivity to oxidative stress, which stimulates iron droplet formation, but also because excess fatty acids (FAs) may enter non-oxidative metabolic pathways, triggering excessive ceramide production [37]. Literature further demonstrates that FAs can be converted into acylcarnitines, leading to mitochondrial fragmentation and dysfunction while generating reactive oxygen species (ROS) [38]. To alleviate this stress, neurons require a mechanism to excrete excess FAs, a process necessitating astrocyte involvement. Lipid metabolism and release between astrocytes and neurons are critical factors in maintaining central lipid homeostasis [8]. We found that PHZ maintains brain lipid homeostasis by promoting astrocytic FA β-oxidation. Co-culturing neurons with astrocytes exhibiting lipid metabolism disorders resulted in decreased neuronal activity, reduced live cell proportion, and significantly increased lipid droplet and triglyceride content in neurons. This indicates that astrocytic lipid metabolism disorders not only affect neuronal activity but also lead to lipid accumulation within neurons. A key limitation of our study is that the co-culture system, while indicative, does not directly prove metabolic transfer. The observed effects could potentially be mediated by other signaling factors or indirect cellular responses. Therefore, our data should be interpreted as supporting a model of metabolic coupling that involves fatty acid transfer, a hypothesis that requires future validation through direct measurement techniques such as tracer-based metabolomics.

Abnormal neuronal lipid transport exacerbates Aβ neurotoxicity. Lipotoxicity impairs synaptic function by disrupting membrane integrity and signaling pathways. Lipid metabolism disorders enhance Aβ production by regulating the APP processing pathway; excess lipids activate the cleavage activity of β-secretase and γ-secretase, leading to abnormal APP hydrolysis and excessive Aβ peptide generation [62]. Our findings also confirm that lipid accumulation in neurons promotes increased expression of secretory enzyme subunits, potentially enhancing Aβ production within neurons. These phenomena were significantly suppressed following PHZ treatment, suggesting PHZ plays a crucial role in regulating central lipid metabolism disorders. Furthermore, abnormal lipid droplet deposition triggered by lipid transport dysfunction activates inflammatory responses in glial cells. Under lipid overload stimulation, microglia release large amounts of pro-inflammatory factors, forming a chronic neuroinflammatory microenvironment [63]. This inflammatory cascade synergizes with Aβ toxicity [64], damaging neuronal metabolic homeostasis and synaptic function, thereby perpetuating a vicious cycle of neurodegenerative pathology [65]. In recent years, significant progress has been made in developing PPAR receptor agonists targeting lipid metabolism abnormalities for AD treatment [66]. Our findings align with other reports demonstrating the beneficial effects of PPARα agonists in AD models [67].

## 5. Conclusions

This study not only identified the role of PHZ in regulating central lipid metabolism disorders but also demonstrated that modulating astrocytic lipid metabolism dysfunction may effectively improve the generation of AD pathological markers and cognitive impairment. PHZ plays a significant role in maintaining normal neuronal function and delaying the progression of AD. Our findings provide novel insights into how PHZ ameliorates AD-associated central lipid metabolism disorders and demonstrate its therapeutic potential for AD.

## Figures and Tables

**Table 1:** Antibodies used, their source, and catalog numbers.

Antibodies	Source	Number
anti-PPARα	Proteintech	66826-1-lg
anti-APOE	Proteintech	66830-1-lg
anti-PS1	Proteintech	16163-1-AP
anti-Pen2	Proteintech	16144-1-AP
anti-BACE1	Proteintech	12807-1-AP
anti-CPT1A	Proteintech	15184-1-AP
anti-GAPDH	Lablead	G1100
Anti-β-tubulin	Lablead	T0100

**Table 2:** Primer sequences used for quantitative real-time PCR (qRT-PCR) analysis.

Gene Target	Gene ID	Forward Sequence	Reverse Sequence	Amplicon Size (bp)
PPARα	19013	AGAGCCCCATATGTCCTCTC	ACTGGTAGTCTGCAAAACCAAA	42
CPT1A	12894	GAACTTGCCCATGTCCTTGT	CCAGGCTACAGTGGGACATT	40
ACSL3	74205	AACCTGTCAGTTCCAAACCG	GCCAATTATAGTGCCCCAGA	40
GAPDH	14433	CGACCACTTTGTCAAGCTCA	AGGGGTCTACATGGCAACTG	40

## Data Availability

The original contributions presented in this study are included in the article.
